# Shoulder Prosthesis

**DOI:** 10.2174/1874325001711011099

**Published:** 2017-09-30

**Authors:** Sebastien Zilber

Shoulder arthroplasty is improving since the late ninetieth century and is still evolving now. Implants, indications, surgical techniques, oversight and survey are changing and need to be updated regularly. This short thematic issue was conducted in this aim.

The exciting history of shoulder arthroplasty is recounted since its beginning and the various trials during the last century. The indications for acute proximal humerus fracture are exposed with the recent development of the use of the reverse arthroplasty. The total anatomic shoulder arthroplasty current surgical techniques were reviewed and analyzed. According to the constant imaging tools improvement, their use for shoulder prosthesis is related. Finally, the early and late complications after shoulder arthroplasty are exposed and should be well known by shoulder surgeon.

This thematic issue is presented by a French and Canadian team strongly involved with shoulder arthroplasty. There are many other papers that could be done about this huge subject. We hope you will like it, and call every interested mind to continue the research.

